# Perirectal Abscess with Anterior Extension to the Extraperitoneum and Space of Retzius: A Case Report

**DOI:** 10.3390/medicina60020293

**Published:** 2024-02-08

**Authors:** Hsiang Teng, Po-Hsien Wu

**Affiliations:** 1 Department of Surgery, Tri-Service General Hospital, National Defense Medical Center, Taipei 114, Taiwan; force22192@gmail.com; 2 Division of Colon and Rectal Surgery, Department of Surgery, Tri-Service General Hospital, National Defense Medical Center, Taipei 114, Taiwan

**Keywords:** perirectal abscess, extraperitoneal extension, space of Retzius

## Abstract

*Introduction*—This report illuminates the distinctive features of a successfully managed Retzius space infection arising from a complex perirectal abscess. It adds novel insights to the scientific literature by addressing the rarity of such occurrences, highlighting the diagnostic complexities associated with extraperitoneal spread, and underscoring the crucial role of a nuanced understanding of anatomy in navigating clinical scenarios involving anorectal abscesses. *Patient’s Main Concerns and Important Clinical Findings*—A 68-year-old male presented with dizziness and diffuse lower abdominal pain, accompanied by intermittent perianal pain for one month. Regardless of an initial misdiagnosis as hemorrhoids, the patient presented sepsis status with fever, hypotension, and tachycardia upon admission. Clinical examinations, including a digital rectal examination, laboratory findings, and imaging studies, revealed a substantial perianal abscess extending into the space of Retzius. Primary Diagnoses, Interventions, and Outcomes—The primary diagnosis involved a heterogeneous fluid-filled perianal abscess extending into the Retzius space, confirmed by abdominal contrast-enhanced computed tomography (CT). Immediate initiation of broad-spectrum antibiotics and subsequent incision and drainage in the 8 o’clock region was performed. Post-operatively, the patient experienced rectal bleeding, necessitating suturing ligation. A follow-up CT scan revealed an extraperitoneal abscess around the bladder, leading to CT-guided drainage and identification of microbial pathogens. Antibiotic treatment with piperacillin-tazobactam was administered. With two weeks of antibiotics and post-operative care, the patient’s symptoms improved, and he was discharged with no signs of recurrence or complications. *Conclusions*—This case report emphasizes the importance of early consideration and identification of extraperitoneal abscesses for timely intervention. The complexity of anatomical planes in extraperitoneal spaces poses diagnostic challenges, necessitating a strategic treatment. The successful management of this case underscores the significance of a multidisciplinary approach, including prompt diagnosis, appropriate antibiotic therapy, and timely surgical interventions, ultimately contributing to improved outcomes in cases involving complex anorectal abscesses.

## 1. Introduction

The classification of anorectal abscesses based on their location includes perianal (60%), ischiorectal (20%), supralevator and intersphincteric (5%), and submucosal (<1%), with perianal abscesses being the most prevalent [1]. Typically, these abscesses arise from anal gland infections, while perirectal abscesses are predominantly caused by cryptoglandular infections located in the small openings in the anal canal lining. On top of that, Approximately 10% of cases are associated with various factors such as trauma, inflammatory bowel diseases like Crohn’s disease or ulcerative colitis, immunocompromised conditions (HIV/AIDS or diabetes), sexually transmitted infections, radiation, foreign bodies, and malignancy [1].

When considering the extension of an abscess beyond the perirectal area into the extraperitoneal space, one should be cautious of infections along fascial planes, including the prevesical space, presacral space, or ischiorectal fossa, as well as adjacent pelvic structures like the uterus, ovaries, or prostate. Among these, spreading across specific anatomical spaces is an infrequent but potentially serious complication, even carrying the risk of mortality. Abscess formation in the space of Retzius is notably uncommon and has primarily been documented in scant case reports [2]. In addition, infections involving the Retzius space are unspecific in clinical presentations, often characterized by patients presenting with vague and atypical symptoms before the identification of a substantial abscess involving various extraperitoneal spaces.

Thus, the complex anatomical planes within the extraperitoneal space provide a conduit for disease dissemination from distant etiologies, posing diagnostic challenges that may hinder the timely implementation of definitive treatments. Also, misdiagnosis or insufficient treatment may result in early recurrence, necessitating a second intervention. In addition, therapeutic strategies vary, influenced by factors such as existing comorbidities or medical histories, which can impact the progress of recovery. Notably, there is a scarcity of previous cases addressing the extraperitoneal spread of perirectal abscess, and a lack of consensus has been established in this regard. Therefore, a comprehensive understanding of anatomy and a heightened awareness of potential management pitfalls are essential when navigating such clinical scenarios. Emphasizing the sharing of experiences through case studies becomes crucial in advancing knowledge and improving patient outcomes.

This report presents a case analysis of an effectively managed Retzius space infection originating from a complex perirectal abscess. In addition, we provide a comprehensive review of the intricacies surrounding the anatomy, pathophysiology, and optimal treatment strategies associated with perirectal abscesses extending into the extraperitoneum. By shedding light on these aspects, this report aims to contribute to the broader knowledge base, facilitating improved clinical management and outcomes in cases involving complex anorectal abscesses.

## 2. Case Description

A 68-year-old male presented to the emergency department with dizziness and diffuse pain located in the lower abdomen over the previous 24 h. He remained awake and alert, with no reported diarrhea, rectal bleeding, blood in stool, or pain in other areas. His medical history revealed hyperlipidemia and hypertension. Notably, there was no history of constipation, long-term diarrhea with abdominal pain, perianal infection, or weight variations. He also denied recent trauma or sexual exposure. However, traced back to his illness from this emergent episode, he had experienced intermittent perianal pain for approximately one month. Initial consultations at local medical clinics had led to an impression of hemorrhoids. Despite prior topical and oral forms of medication, the pain persisted and discomforted him for a while, leading to his current emergent episode.

This time, upon admission, he was febrile (temperature: 38.5 °C), hypotensive (blood pressure: 96/62 mmHg), and slightly tachycardic (heart rate: 102 beats/min), necessitating immediate administration of inotropic agents. A physical examination revealed dull pain with deep palpation over the groin and suprapubic area, without rebounding pain or signs of deep vein thrombosis in the extremities. Notably, the peritoneal sign was negative at that time. A digital rectal examination did not reveal bloody discharge, but diffuse and nonspecific pain was noted. However, there were no alarming symptoms impacting ambulation or normal gait, and the strength of extremities was not impaired either. The patient did not report dysuria or increased urinary frequency.

**Figure 1:**
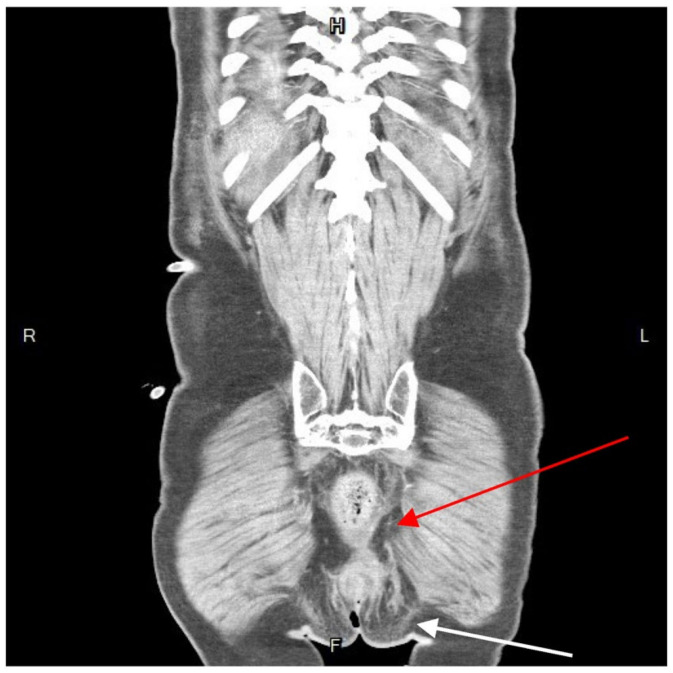
Coronal view of contrast-enhanced computed tomography. A hypo-attenuated area (size: 5.0 × 4.4 × 6.8 cm) on the left side perianal, with spotted air and irregular contour (arrow). The abscess cavity showed contralateral extension to the supra-sphincteric direction (red arrow). R, right; L, left; H, superior; F, inferior.

**Figure 2:**
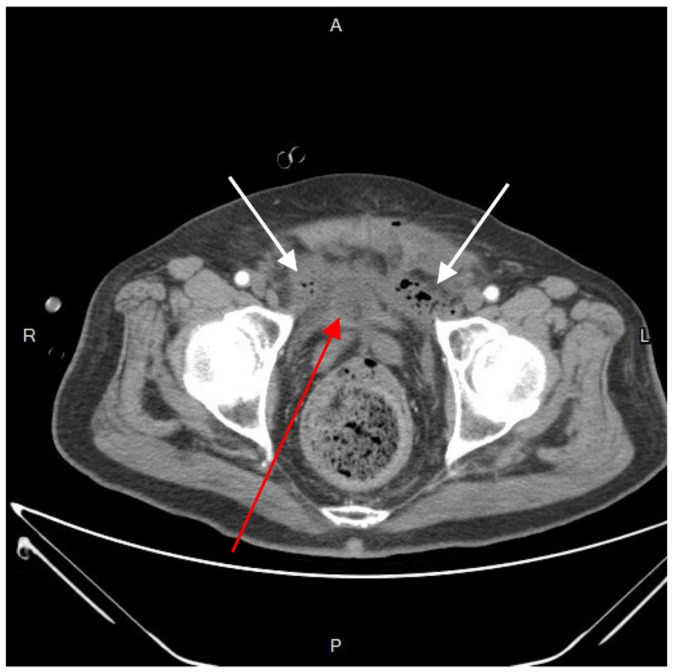
Axial view of contrast-enhanced computed tomography. An emphysematous collection of fluid-and-air-filled cavity (white arrow) in the perivesical space, dominantly on the left side. A Foley catheter with inflated balloon was dwelled in the bladder (red arrow). R, right; L, left; A, anterior; P, posterior.

**Figure 3:**
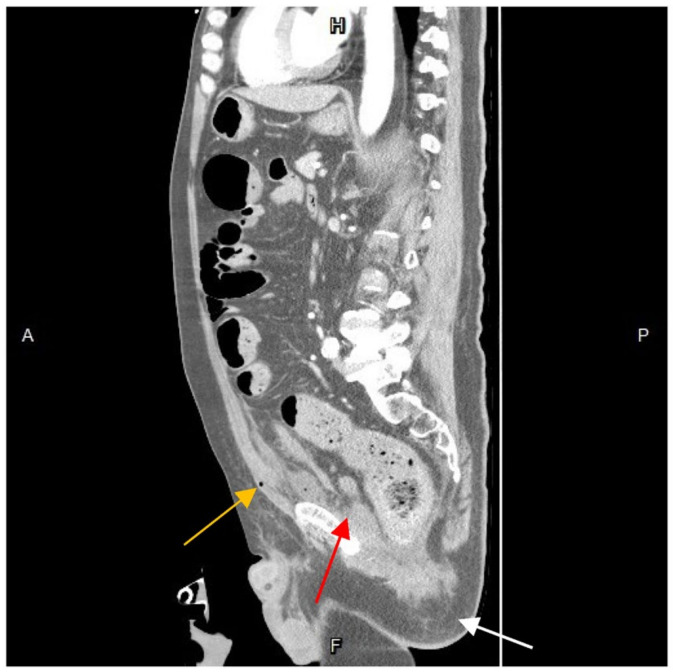
Sagittal view of contrast-enhanced computed tomography. The abscess formation seemed to originate in the perianal region (white arrow) and migrate to the supra-levator plane (red arrow) and the preperitoneal space (yellow arrow). With close inspection, the air is spotted extraperitoneal. A, anterior; P, posterior; H, superior; F, inferior.

Further diagnostic measures included a plain abdominal radiograph, which showed no pneumoperitoneum or abnormal fluid accumulation. Laboratory findings consisted of an elevated white blood cell count and C-reactive protein level (white blood cells: 27,830/μL; neutrophils: 90.6%; C-reactive protein: 21.65 mg/L). Abdominal contrast-enhanced computed tomography (CT) showed a heterogenous fluid-filled perianal abscess measuring 5.0 × 4.4 × 6.8 cm (Figure 1) in the left perianal region, with extraperitoneal air spreading through the abdominal wall fascia (Figure 2 and Figure 3). The primary abscess was situated in the supra-levator space, potentially involving the levator muscle (infra-levator), as indicated by its migration from the perianal level to the extraperitoneal level. After a comprehensive review of the patient’s symptoms, medical history, age, and current status, immediate arrangements for endoscopic tests, including upper gastrointestinal endoscopy and colonoscopy, were not made. Additionally, gastrointestinal tract specimens, such as fecal samples for calprotectin and lactoferrin, were not collected, thereby not suggesting the likelihood of inflammatory bowel diseases.

Blood cultures were drawn, and broad-spectrum empiric antibiotics (meropenem) were initiated. Following fluid resuscitation, the patient underwent incision and drainage in the 8 o’clock region. A cystic-like cavity filled with pus and surrounded with granuloma was found. We debrided and irrigated the lesion as much as possible and afterwards placed a Foley tube for adequate drainage and irrigation. Subsequent dressing changes were conducted regularly for proper hygiene. However, ten days after the surgery, the patient experienced sudden massive rectal bleeding, resulting in hypovolemic shock. Upon examination with an anal retractor, an oozing site with active bleeding was identified in the rectum in the 9 o’clock region. On the spot, the bleeding was managed by suturing ligation with 2-0 Vicryl successfully. Meanwhile, a colonoscopy revealed no obvious bleeding, mucosal constriction, fistula, rectal ulcers, or pre-malignant abnormalities. Simultaneously, an emergent abdomen and pelvic CT scan indicated no contrast medium extravasation in the bowel lumen or peritoneal space. Still, it identified an irregularly thick wall at the right side of the rectum (1.85 × 5.9 cm) and an extraperitoneal abscess around the bladder. Despite these findings, an anal-rectal fistula was not observed based on image surveys and physical examinations.

**Figure 4:**
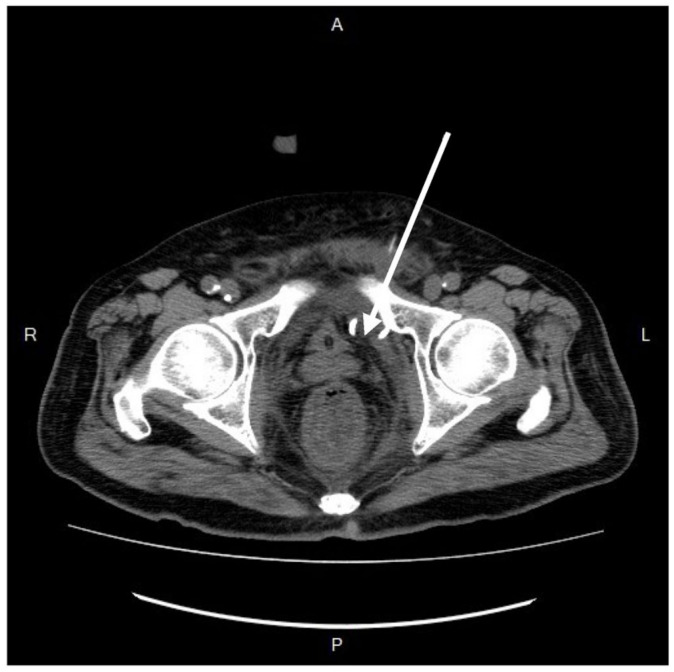
A computed-tomography-guided percutaneous drainage procedure. The arrow demonstrated the direction and position of needle insertion. R, right; L, left; A, anterior; P, posterior.

After resuscitation and blood transfusion, the patient’s vital signs stabilized, and the inotrope support was tapered. The next day, as confirmed by the CT scan and concerned about the further progression of infection, we consulted a radiologist for CT-guided extraperitoneal abscess drainage (Figure 4) and collected a significant amount of turbid fluid. Cultures from the pus were used to identify *Klebsiella pneumoniae*, *Escherichia coli*, *Viridans streptococcus* gr., and *Bacteroides fragilis*. Subsequently, antibiotic treatment with piperacillin-tazobactam was prescribed accordingly.

Within two weeks of antibiotics treatment and post-operative care, his hypogastric pain and fever improved gradually. The patient became afebrile with normal laboratory values, allowing him to be discharged from the hospital. During the 3-month follow-up in the out-patient department, a digital rectal examination revealed no apparent fistula tract formation, and the wound was healed with normal anal tonicity. There were no signs of recurrence or long-term complications.

## 3. Discussion

The space of Retzius, also known as the prevesical or retropubic space, is delineated externally by the transversalis fascia and internally by the parietal peritoneum. Its lateral extension includes the space of Bogros below the inguinal ligament, housing iliofemoral vessels and iliopsoas muscles. Continuing posterolaterally, it is directly contiguous with the infrarenal retroperitoneal compartments and caudally extends to the supra-levator extraperitoneal space [3]. Also, anatomically, the supra-levator space is a pelvic compartment above the levator ani muscle that communicates anteriorly with the space of Retzius, bilaterally with the retro-inguinal spaces, and posteriorly with the retroperitoneum. The puborectalis muscle may act as a barrier to inferior abscess expansion; although, aggressive invasion may occur, facilitating infections spread through the anterior and posterior extraperitoneal abdominal compartments.

Via the results of a perfusion study and cadaver dissection, Chen et al. [4] demonstrated communication between the pararectal spaces and the vesical extraperitoneal space anteriorly. Owing to the umbilical–vesical fascia ending at the reflection of the vesical peritoneum, the bellowing adipose tissue connects between the side of the vesica and the pararectal space.

Moreover, the space of Retzius is typically characterized by fat, loose fibrous tissue, and a perivesical venous plexus. Although abscess formation in this space is relatively rare, cases have been reported with various etiologies, such as adjacent contacts or indirect spreading from nearby organs, including bladder infections, septic arthritis or osteomyelitis of the pubic symphysis, perianal abscesses, and Fournier’s gangrene. The bacterial etiology of an abscess often depends on the original inciting infection. For instance, a perianal abscess extending to the prevesical space would more likely be secondary to a polymicrobial infection including Gram-negative anaerobes.

In our patient’s case, the original perirectal abscess likely extended cephalad through the supralevator space, progressed into the iliac space, and then advanced lateral to medial into the prevesical space.

From the aspect of treatment, the therapeutic strategies vary, with percutaneous drainage being the preferred method. Meanwhile, open surgical treatment and debridement may be necessary and inevitable, especially in cases of widespread abscesses and patients in septic states. The reported cases have demonstrated several managements [5]. The optimal treatments include extraperitoneal drainage with a lower midline abdominal incision [6] and additional fistulectomy and diverting loop sigmoidostomy [7]. The wound can be closed primarily with drains or vacuum-assisted devices [8]. Notably, in a massive abscess with extraperitoneal extension, access to the peritoneal cavity and transperitoneal manipulation must be avoided due to the high risk of contamination and secondary peritonitis. On the contrary, if an abscess is unilocular, relatively free of heterogenous particulate matter, and safely approachable, percutaneous drainage of the abscess seems to be a favorable maneuver. Surgical debridement can always be instituted later if sepsis is not controlled prominently.

In our patient’s case, upon the patient’s arrival in the emergency department, he reported mild dull pain in the lower abdomen region and demonstrated a negative peritoneal sign during the physical examination. The digital rectal examination revealed diffuse palpable pain without specific direction. The CT scan also identified a hypo-attenuated area in the left perianal region, with no evidence of fluid accumulation or gas formation in the peritoneal cavity. Given the impression of a perianal abscess, debridement was indicated. Post-operatively, the patient’s septic status and infectious condition improved under initial conservative management, including wound care with frequent irrigation and dress changing.

In retrospect, given the spreading abscess noted around the bladder via CT scan and fluctuating blood pressure levels, diagnostic laparotomy over the lower abdomen should have been considered. However, during an emergent CT scan conducted following an episode of anal bleeding, a progressed extraperitoneal abscess was identified, located anteriorly to the bladder. It was also reported that after the primary incision, early recurrence was seen as a major risk factor for abscess expansion, leading to worse outcomes [5]. Accordingly, secondary intervention should involve more thorough and adequate strategies. Nevertheless, at this point, the abscess was primarily contained in the extraperitoneal space without causing extensive invasion leading to systemic instability. The radiologist also suggested that the pre-vesical abscess was approachable and drainable. Consequently, based on previous experience and the radiologist’s suggestion, percutaneous drainage was selected as the intervention and was successfully performed.

Moreover, upon abscess detection, blood cultures should be drawn, and broad-spectrum antibiotics, such as vancomycin and piperacillin-tazobactam or a combination of vancomycin, cefepime, and metronidazole should be administrated early. The antibiotic regimen should cover methicillin-resistant *Staphylococcus aureus* and Gram-negative anaerobes, considering possible hematogenous seeding and the contiguous spread of infection. A prolonged course may be required if accompanying osteomyelitis is present.

Deep-spreading perianal abscesses, particularly those with extraperitoneal invasion, puzzle physicians with diagnostic challenges due to their rarity and indolent clinical manifestations. Abdominal pain is indicative of an extraperitoneal spreading abscess in a large portion of patients (86%) with that presentation [5]. Associated medical illnesses, such as diabetes mellitus, alcoholism, end-stage renal disease, and immunocompromization, could predispose patients to higher complication rates, with pneumonia and respiratory failure being common [9]. That is, a baseline frail status coupled with an extensive infection may accelerate disease progression and severity. Furthermore, the number of days for fever to subside after a drainage procedure is a significant prognostic indicator related to the mortality rate [9]. Patients with preoperative fevers where the temperature fell below 100 °F in less than 3 days had an 89% mortality rate, compared to 71% in those who remained febrile (*p* < 0.05) [9]. Meanwhile, the overall mortality rate for extraperitoneal abscesses ranges from 22% to 46%. If a patient does not show rapid clinical improvement after initial drainage, a clinician must remain vigilant for signs of a complicated condition. With increasing surgical manipulation in complex extraperitoneal abscesses, surgeons must be familiar with their potential interconnections, considering both sources and interventions. With earlier diagnosis, more immediate surgical drainage facilitated by CT imaging should decrease the related morbidity and mortality risks.

## 4. Conclusions

This is a unique case of a septic patient diagnosed with a perirectal abscess with upward extension to the extraperitoneal space. Early consideration and identification of extraperitoneal abscesses are pivotal for timely intervention. A more accurate and efficient diagnosis and treatment could reduce derived complications, reducing the risks of morbidity and mortality.

## Data Availability

The original contributions presented in the study are included in the article, further inquiries can be directed to the corresponding author.
